# Regulation of ErbB Receptors by the Ca^2+^ Sensor Protein Calmodulin in Cancer

**DOI:** 10.3390/biomedicines11030661

**Published:** 2023-02-22

**Authors:** Antonio Villalobo

**Affiliations:** Cancer and Human Molecular Genetics Area—Oto-Neurosurgery Research Group, University Hospital La Paz Research Institute (IdiPAZ), Paseo de la Castellana 261, E-28046 Madrid, Spain; antonio.villalobo@idipaz.es

**Keywords:** calcium, calcineurin, calmodulin, calmodulin-dependent kinases, epidermal growth factor receptor, ErbB receptors

## Abstract

Overexpression and mutations of the epidermal growth factor receptor (EGFR/ErbB1/HER1) and other tyrosine kinase receptors of the ErbB family (ErbB2/HER2, ErbB3/HER3 and ErbB4/HER4) play an essential role in enhancing the proliferation, the migratory capacity and invasiveness of many tumor cells, leading to cancer progression and increased malignancy. To understand these cellular processes in detail is essential to understand at a molecular level the signaling pathways and regulatory mechanisms controlling these receptors. In this regard, calmodulin (CaM) is a Ca^2+^-sensor protein that directly interacts with and regulates ErbB receptors, as well as some CaM-dependent kinases that also regulate these receptors, particularly EGFR and ErbB2, adding an additional layer of CaM-dependent regulation to this system. In this short review, an update of recent advances in this area is presented, covering the direct action of Ca^2+^/CaM on the four ErbB family members mostly in tumor cells and the indirect action of Ca^2+^/CaM on the receptors via CaM-regulated kinases. It is expected that further understanding of the CaM-dependent mechanisms regulating the ErbB receptors in future studies could identify new therapeutic targets in these systems that could help to control or delay cancer progression.

## 1. Introduction

Mutations and the overexpression of the tyrosine kinase ErbB (erythroblastic leukemia viral oncogene homolog) receptor family members (EGFR/ErbB1/HER1, ErbB2/HER2, ErbB3/HER3 and ErbB4/HER4) have been shown to play a prominent role in oncogenesis; in particular, the epidermal growth factor receptor (EGFR) and ErbB2, also denoted as human EGFR-2 (HER2), contribute to the enhanced proliferative capacity, promotion of cell survival, development of tumor-associated angiogenesis and increased migratory and invasive capacities of tumor cells. These processes lead to the development of metastases in distant organs, which is the primary cause of mortality. All the regulatory mechanisms of these receptors have not been extensively studied, but they have been targeted to treat different human cancers in the clinic (reviewed in [1,2,3,4,5]).

Of interest for our subject topic, the Ca^2+^-sensor and transducer protein calmodulin (CaM), directly upon interaction with the ErbB receptors and indirectly, for example, by phosphorylation of the receptors via CaM-dependent kinases, regulate their activity and functions. In addition, it is known that these CaM-regulated systems play many functional roles in cancer (reviewed in [6]). In humans and other mammals, three distinct genes in different chromosomes code for a CaM protein with an identical sequence. These CaM genes (*CALM1*, *CALM2* and *CALM3*) are differentially expressed in the cell using different codons and mRNAs with distinct stability (reviewed in [7]). This apparent redundancy is not only a safeguard for cell survival, but the different codon usage results in the differential expression of each gene in a given organ at the various stages of development and functionality. CaM interacts with and regulates the function of hundreds of proteins using different modes (reviewed in [8]), and it is also able to act as an adaptor protein, bridging either different domains of a target protein to generate a functional domain modulating its activity or linking two distinct proteins contributing to their functionality in the cell (reviewed in [9]). In addition, to be modulated by Ca^2+^ binding to the two pairs of EF hands in the N- and C-lobes at variable saturation levels, CaM also regulates some proteins in its Ca^2+^-free form binding to the so-called IQ domains (reviewed in [10]). CaM can be phosphorylated by Ser/Thr- and Tyr-kinases, and their phosphorylated forms regulate in a distinct manner compared to non-phosphorylated CaM a variety of target proteins (reviewed in [11,12]). The most exciting development in recent years in the CaM field has been the discovery of naturally occurring mutants of CaM in humans, implicating all of its three genes, that have been shown to affect the functionality of some target proteins, including ion channels, inducing their malfunction and serious illness in the heart and possibly other organs, denoted as calmodulinopathies (reviewed in [7,13,14,15,16]).

Of medical interest is also the implication of Ca^2+^/CaM in neurological disorders, such as Alzheimer’s disease, where different CaM-binding proteins (CaM-BPs) are involved in amyloidogenic pathways and the amyloid-β protein is also a CaM-BP, contributing to the onset and progression of the disease (reviewed in [17]). The implication of CaM is also extended to other neurodegenerative processes, such as Parkinson’s, Huntington’s, Lewy bodies and amyotrophic lateral sclerosis/frontotemporal diseases, where many CaM-BPs are implicated, and their alterations are considered risk factors (reviewed in [18]).

More than a decade ago, we published some reviews describing the major features of the regulation of the epidermal growth factor receptor (EGFR) and its family member ErbB2 by CaM [19,20]. Therefore, in the present minireview, besides briefly mentioning some early work to better understand the evolution of this field, emphasis will be focused on more recent advances in this area, particularly in cancer. We now describe the latest works on the effect of CaM on the four ErbB receptor family members, and the regulation of these receptors by other CaM-dependent systems (e.g., CaM-dependent kinases).

## 2. Regulation of the EGFR by CaM

**Figure 1:**
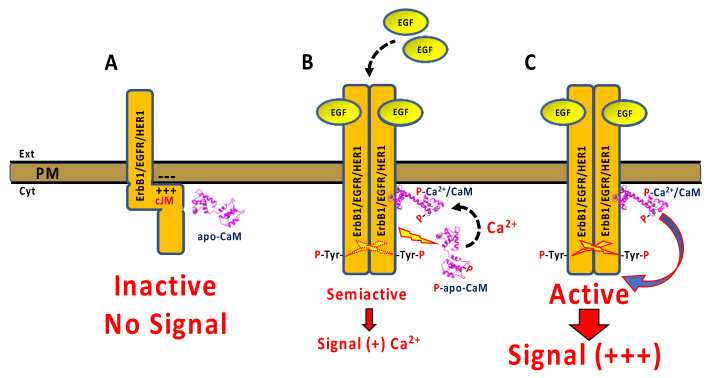
Model of EGFR activation by the cooperative action of EGF and the phospho- Ca^2+^-CaM complex. The cartoon shows (**A**) the ligand-free inactive EGFR, in which the cytosolic juxtamembrane region (*cJM*) is electrostatically bound to the inner leaflet of the plasma membrane (*PM*) inhibiting the receptor [25], and Ca^2^-free CaM (*apo-CaM*) is near to the receptor. (**B**) Binding of the ligand EGF induces EGFR dimerization, a weak activation sufficient to phosphorylate apo-CaM [30,31] and induce a Ca^2+^ transient that forms the Ca^2+^/CaM complex, which binds to the *CaM-BD* at the *cJM* that is released from the inner leaflet of the *PM*. (**C**) EGF plus phospho-Ca^2+^/CaM induce the full activation and signaling of the receptor. The structures of Ca^2+^-bound CaM (PDB ID: 1CLL) [32] and apo-CaM (PDB ID: 1DMO) [33] were taken from the National Center for Biotechnology Information (NCBI). Apo-CaM, Ca^2+^-free calmodulin; Ca^2+^/CaM, Ca^2+^-bound CaM; CaM-BD, CaM-binding domain; cJM, cytosolic juxtamembrane region; Cyt, cytosol; EGF, epidermal growth factor; EGFR, EGF receptor; Ext, extracellular medium; PM, plasma membrane.

The direct interaction of Ca^2+^/CaM with the EGFR from rat liver, and the identification of its CaM-binding domain (CaM-BD) at its cytosolic juxtamembrane (cJM) region was first established by our group [21,22] and confirmed by others [23,24,25]. This interaction occurs at the highly basic sequence RRRHIVRKRTLRRLLQ (residues 645–660) of the human receptor. To clarify the functional role of this interaction, essential work was later performed using a peptide corresponding to the transmembrane plus the CaM-BD regions (residues 622–660) incorporated into the membrane of artificial lipid vesicles, in which the CaM-BD segment bends and binds to the membrane surface rich in acidic lipids. The addition of Ca^2+^/CaM released the CaM-BD from the membrane surface [26] (see Figure 1), hence confirming the electrostatic engine model for the autoinhibition of the EGFR earlier proposed by McLaughlin and co-workers [25], preventing the uncontrolled activation of the receptor in the absence of ligand. The mechanism of EGFR activation by forming an allosteric asymmetric dimer [27] and the essential role of its cJM region where the CaM-BD is located in helping to dimerize the kinase domain of the two monomers forming the active dimer, was stablished by the Carpenter [28] and Kuriyan [29] groups.

The guanine nucleotide exchange factor ARNO (ARF nucleotide-binding site opener) [34], via its central Sec7 domain (homologous to the yeast protein Sec7p), interacts in vitro with the cJM region of the EGFR essentially at the same site that Ca^2+^/CaM does [35]. In fact, competition between ARNO and CaM for the binding to this site has been demonstrated [35], suggesting to the authors of this work that both proteins may have a similar activating role on the EGFR, separating, upon binding, the positively charged CaM/ARNO-binding site from the phosphoinositide-rich highly negative-charged inner leaflet of the plasma membrane, as proposed by McLaughlin and co-workers [25].

**Figure 2:**
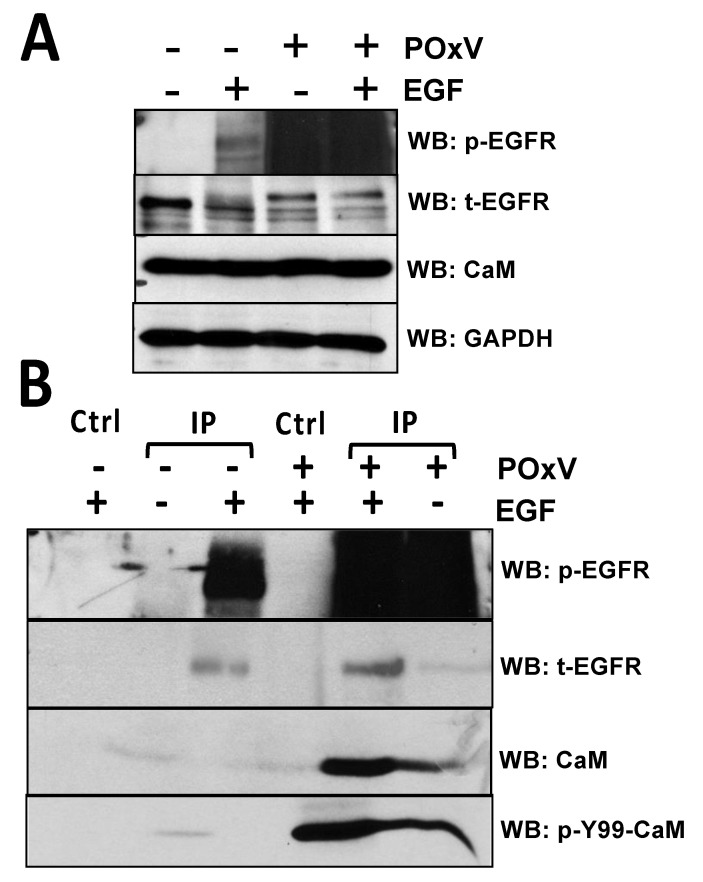
The EGFR phosphorylates CaM. (**A**) HeLa cells were treated in the absence and presence of 1 mM peroxovanadate (POxV), a cell permeable tyrosine phosphatase inhibitor and thereafter stimulated with 20 nM EGF for 5 min. The level of phospho-EGFR (*p-EGFR*), total EGFR (*t-EGFR*) and CaM were determined using Western blot (*WB*) in the total cell extract with a monoclonal (rabbit IgG) anti-EGFR antibody (clone E235) from Millipore recognizing the C-terminal region, a monoclonal (mouse IgG_2bκ_) anti-phospho-tyrosine antibody (clone 4G10) from Upstate Biotechnology recognizing anti-phospho-EGFR and a monoclonal (mouse IgG_1_) anti-CaM antibody from Millipore, respectively. GADPH, determined using a monoclonal (rabbit IgG) anti-GAPDH antibody (clone 14C10) from Cell Signaling Technology, is also shown as a loading control. The presence of POxV induced an extraordinary phosphorylation of the EGFR by preventing dephosphorylation, which results in the unavoidable overexposure of the X-ray film. (**B**) HeLa cells treated in the absence and presence of POxV and EGF, as in panel *A*, were lysed and immunoprecipitated (IP) with an anti-phospho-tyrosine antibody. Non-relevant protein A-agarose beads were used as control (Ctrl). Phospho-EGFR (*p-EGFR*), total EGFR (*t-EGFR*), CaM and phospho-tyrosine99-CaM (*p-Y99-CaM*) were determined using Western blot (*WB*) with anti-phospho-tyrosine, anti-EGFR, anti-CaM and anti-phospho-Tyr99-CaM antibodies, respectively (Sánchez-González and Villalobo, additional data to [30,31,37]).

The interaction of CaM with the EGFR in keratinocytes was also established when studying the CaM/cadherin/EGFR signaling pathway [36]. In this work, using an anti-CaM antibody, the EGFR was co-immunoprecipitated, but a phospho-EGFR was not detected. However, the phosphorylated (active) EGFR was detected when immunoprecipitated using an anti-phosphoTyr138-CaM antibody [36]. This suggests that the interaction of CaM with the EGFR first occurs with the non-active receptor, and the receptor is partially activated by the coordinated action of its ligand (e.g., EGF) and non-phosphorylated Ca^2+^/CaM, which detaches the juxtamembrane region of the receptor from the inner leaflet of the membrane [25]. Thereafter, the partially active EGFR phosphorylates CaM [30,31,37], which further enhances its activity, in agreement with our findings in human carcinoma epidermoid A431 cells [38] (reviewed in [12]). To document the phosphorylation of CaM by the EGFR, we show in Figure 2 an additional example performed in live human cervix carcinoma HeLa cells, complementing our previous reports carried out with the isolated receptor from rat liver and detergent-permeabilized fibroblasts overexpressing the EGFR [30,31,37]. Once the CaM-BD is detached from the membrane, we have proposed that this positively charged amphiphilic basic segment binds electrostatically to a negatively charged acidic region of the receptor at the sequence DEEDMDDVVDADEY (residues 979–972) located downstream of the tyrosine kinase domain and denoted as the CaM-like domain (CaM-LD) for its similarity to a region of CaM at its C-terminus (residues 118–130 with sequence DEEVDEMIREADI) [39,40].

To delve further into the functional importance of CaM-BD, further studies were conducted using transfectants with deletion mutants of the EGFR, lacking the CaM-BD or by substituting the basic residues for neutral ones. It was established that the tyrosine kinase of these mutants was inactive [40,41,42], although we demonstrated in our system that the receptor was able to bind EGF but not to be internalized [40]. On the other hand, deleting the CaM-LD or altering its sequence inserting in its place the valine/histidine-rich peptide LEGIHHVVHVFFLE, it was determined that both mutant receptors bind EGF, but only the deletion mutant, and not the one with the altered sequence, retained some tyrosine kinase activity, although both of them were internalized [40]. These puzzling observations require further investigation to understand the reason for the distinct behavior of both mutants in terms of internalization in the absence of trans(auto)phosphorylation of one of them.

Using a technique based on fluorescent polarization, it was established that the four EF hands of CaM must be occupied with Ca^2+^ to interact with the peptide corresponding to CaM-BD of the EGFR [24]. In contrast, and as an example of different CaM saturation requirements acting on other target proteins, binding of only two Ca^2+^ sufficed for CaM to interact with a peptide corresponding to the CaM-BD of DAPK (death-associated protein kinase) [24] or to induce basal activity and inactivation of the L-type voltage-regulated Ca^2+^ channel Ca_v_1.2 [43], giving credit to the hypothesis that a transient increase in the cytosolic concentration of Ca^2+^ of a high magnitude should occur to saturate CaM with Ca^2+^ to elicit its action on the EGFR, in contrast with the lower magnitude Ca^2+^ transients required to regulate other target proteins.

In two transformed murine fibroblast cell lines, stable transfectants overexpressing the human EGFR, mimicking the overexpression of this receptor observed in many carcinomas, it was observed that a myristoylated myosin light-chain kinase (MLCK) peptide, which sequesters endogenous CaM or the CaM antagonists W-7 (*N*-(6-aminohexyl)-5-chloro-1-naphthalenesulfonamide) and W-13 (*N*-(4-aminobutyl)-5-chloro-2-naphthalenesulfonamide), inhibited the EGF-dependent activation of the receptor [44]. Moreover, similar results were obtained in human breast adenocarcinoma SK-BR-3 cells, which overexpress ErbB2 instead of EGFR [44]. In addition, chelating intracellular Ca^2+^ with BAPTA-AM (1,2-bis(2-aminophenoxy)ethane-N,N,N’,N’-tetraacetic acid tetrakis [acetoxymethyl ester]) in human neuroblastoma NB69 cells and human lung adenocarcinoma A549 cells inhibited the EGF-dependent activation of the receptor [44]. Overall, these results suggest that Ca^2+^/CaM acts as an intracellular co-activator of the EGFR and ErbB2, facilitating the action of the extracellular ligands; therefore, it could be considered a potential therapeutic target. CaM antagonists have been shown to potentiate the action of different chemotherapeutic agents in many tumor cells in culture (reviewed in [6]). However, the information on the use of CaM inhibitors in the clinic to treat cancer is very scanty, perhaps due to the fact that CaM controls many cellular processes, and unwanted side effects are unavoidable. Nevertheless, some clinical trials have been performed, particularly using the CaM antagonist trifluoperazine in combination with the anti-tumor DNA-binding agents bleomycin and doxorubicin to treat cancer patients, showing some potentiation of the chemotherapeutic agents used, but some toxic effects were also detected, although the authors emphasized that the treatment was well tolerated [45,46,47,48]. In any event, it is very unlikely that the effect of this CaM inhibitor was exclusively centered in the disruption of the CaM/EGFR interaction, as no information on this is available.

**Figure 3:**
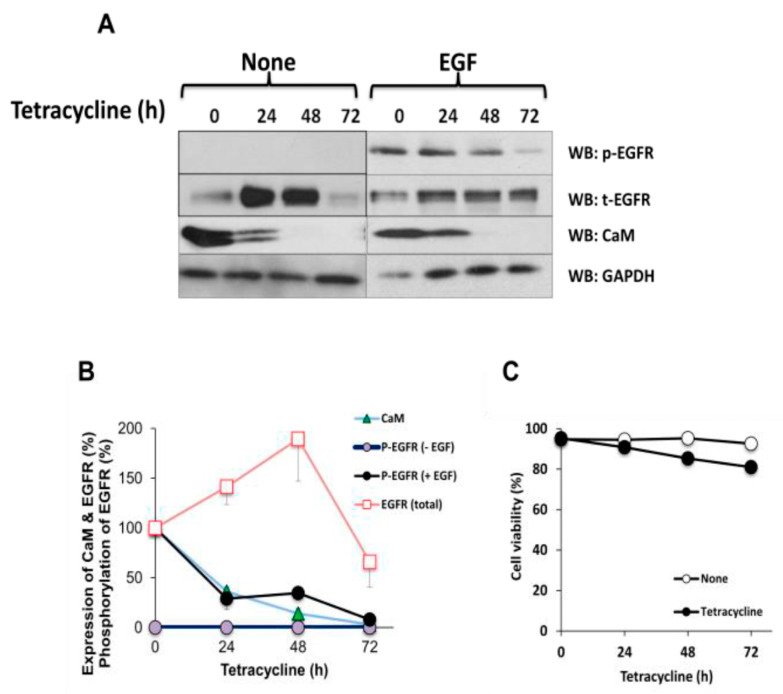
CaM downregulation in conditional CaM-KO cells decreases EGF-dependent EGFR activation. (**A**) ET1-55/EGFR cells were incubated with 1 μg/mL tetracycline (TET) for the indicated times and stimulated with 10 nM EGF for 5 min. The expression of EGFR (*t-EGFR*), phospho-EGFR (*p-EGFR*) and CaM were determined using a monoclonal (rabbit IgG) anti-EGFR antibody (clone E235) from Millipore recognizing the C-terminal region, a monoclonal (mouse IgG_2bκ_) anti-phospho-tyrosine antibody (clone 4G10) from Upstate Biotechnology recognizing anti-phospho-EGFR and a monoclonal (mouse IgG_1_) anti-CaM antibody from Millipore, respectively. GADPH, determined using a monoclonal (rabbit IgG) anti-GAPDH antibody (clone 14C10) from Cell Signaling Technology, is also shown as a loading control. (**B**) The plot presents the mean ± SEM (n = 5 for total EGFR, n = 3 for phospho-EGFR, and n = 3 for CaM) expression of total EGFR (red squares), phospho-EGFR in the absence (gray circles), presence (black circles) of EGF and CaM (green triangles) in the presence of TET for the indicated times. (**C**) The plot represents the mean ± SEM (n = 3) cell viability measured by trypan blue exclusion test in the absence and presence of TET for the indicated times (Stateva and Villalobo, additional data to [44]).

The development of conditional CaM-knockout (CaM-KO) cell lines using tetracycline (TET)/doxycycline (DOX)-off systems by Berchtold and colleagues [49,50] opened the possibility to study the action of Ca^2+^/CaM on the ligand-dependent activation of the EGFR in better detail [44,51]. In this context, it was demonstrated that in conditional CaM-KO chicken pre-B lymphoma DT40 cells (clone ET1-55) stably transfected with the human EGFR, the addition of TET induced a time-dependent downregulation of the cellular levels of CaM, which was accompanied by the progressive inhibition of the EGF-dependent activation of the receptor [44]. This occurs without significant effects on cell viability or the intracellular ATP levels in the first 72 h, as in wild-type DT40 cells stably transfected with the human EGFR [44]. Figure 3 shows an example of EGFR inactivation upon CaM downregulation in CaM-KO DT40 cells. A surprising new result was the transient increased expression of the non-active EGFR at 24–48 h upon tetracycline-induced CaM downregulation. The reason for this effect is unknown, although one possibility is that the basal CaM levels were somehow inhibiting the translation of the EGFR and that this inhibition disappears upon CaM downregulation during this limited time frame; this possibility should be investigated in the future.

In conditional CaM-KO HeLa cells prepared by the CRISPR/Cas9-mediated deletion of the three endogenous CaM genes and expression of an ectopically inserted rat CaM coding sequence, the downregulation of CaM upon addition of DOX failed to induce any decrease in ligand-dependent EGFR auto(trans)phosphorylation/activation. This is in contrast to the results obtained in CaM-KO DT40 cells stably transfected with the human EGFR [44]. We speculated that the observed lack of response to CaM downregulation in HeLa cells could be due to high protein kinase C (PKC)-mediated phosphorylation of the EGFR at Thr654 [51], as we previously demonstrated that phosphorylation of Thr654 by PKC prevents CaM binding to the CaM-BD of the EGFR [22], and it has been established that HeLa cells have a high number of low-affinity receptors (more than 50% of total EGFR molecules) [52], likely due to the phosphorylation of Thr654 by PKC, as the addition of phorbol esters induced the conversion of high-affinity to low-affinity receptors [53,54]. Curiously, a significant decrease in the expression of the receptor in the presence of EGF was detected. This was also observed, although to a lesser extent, in control wild-type HeLa cells [51]. The reason for this behavior is unknown at present.

Moreover, CaM downregulation in conditional CaM-KO HeLa cells inhibited cell migration in a 2D surface (wound healing assays) in the absence but not in the presence of EGF, while the inhibition of cell migration in a 3D system (porous membrane assays) was observed upon CaM downregulation, both in the absence and presence of EGF [51]. This suggests that some obligatory CaM-dependent system(s) is (are) operative during 2D migration in the absence of EGF but not during EGF-dependent migration. In contrast, some essential CaM-dependent pathway(s) is (are) absolutely required for 3D migration, both in the absence and presence of EGF [51].

Increasing the cytosolic Ca^2+^ level facilitates the formation of the Ca^2+^/CaM complex required for ligand-dependent EGFR activation. In addition to CaM-modulated Ca^2+^ channels—for example, the inositol 3-phosphate receptor (IP_3_R) and the calcium release-activated calcium channel protein 1 (Orai1) regulated by the stromal interaction molecule 1 (Stim1)—which are opened, facilitating the replenishment of cytosolic Ca^2+^ when required, other CaM-regulated systems are operative, such as the Ca^2+^-sensitive chloride channel accessory protein 2 (CLCA2), which activates Orai1,and the CaM-regulated chloride channel denoted as transmembrane member 16A/anoctamin 1 (TMEM16A/ANO1) [55], which activates the EGFR and downstream signaling pathways (reviewed in [56]). These accessory CaM-modulated systems are deregulated in some tumor cells, affecting important cellular functions (reviewed in [56]). As an example, TMEM16A/ANO1 appears amplified and overexpressed in breast cancer, contributing to enhanced malignancy and proliferative activity via EGFR signaling pathways [57]. In fact, knocking-down or pharmacological inhibition of TMEM16A/ANO1 in tumor cells inhibited their proliferation by reducing EGFR activation and its downstream signaling pathways (reviewed in [58]).

A missing point in the study on the interaction of CaM with the EGFR and other ErbB receptors in live cells and its relation to oscillations of the Ca^2+^ signal could be solved using a combination of FRET (Föster resonance energy transfer)/BRET (bioluminescence resonance energy transfer)/FLIM (fluorescence lifetime imaging microscopy) technologies in stably transfected cells expressing genetically encoded marked CaM and receptors marked with no interfering spectral-compatible indicators (reviewed in [59,60,61,62]). This can be complemented by the simultaneous use of FRET/BRET-based biosensors to detect the transient increase in the cytosolic Ca^2+^ concentration by the activation of the receptors upon ligand binding [63,64]. To avoid interferences with endogenous CaM and receptors, these experiments should be performed in engineered conditional CaM-KO cells, as previously described [49,50], lacking the receptors under study after endogenous CaM downregulation.

## 3. Regulation of Other ErbB Receptors by CaM

Given the structural or functional similarities between the different members of the human tyrosine kinase ErbB receptor family, it was expected that in addition to the EGFR, other ErbB family members could directly interact with Ca^2+^/CaM. In an early report, it was demonstrated that peptides corresponding to the cytosolic juxtamembrane region of the four ErbB receptors bind CaM with different affinities, presenting Ca^2+^/CaM association constants of 0.01, 0.2, 0.6 and 3 μM for the EGFR (645–660), ErbB4 (676–692), ErbB2 (676–692) and ErbB3 (667–683) peptides, respectively [25]. The peptide that presents the lowest affinity for Ca^2+^/CaM is the one from ErbB3, which lacks tyrosine kinase activity (reviewed in [65]), suggesting that the binding of Ca^2+^/CaM may be irrelevant for the activation of this receptor in contrast to the EGFR and ErbB2, and possibly ErbB4, although information about the latter is lacking at this time.

In this context, we and others demonstrated that, indeed, Ca^2+^/CaM binds to and regulates ErbB2 and its downstream signaling pathways, as CaM inhibition or deletion of the CaM binding sites inhibit ErbB2-mediated signaling, decreasing tumor cells proliferation [66,67]. Ca^2+^/CaM was shown to bind to two sites, one at the cytosolic juxtamembrane region, equivalent to the CaM-binding site in the EGFR and another downstream of the first one, as deleting residues 676–689 or 714–732 both prevented Ca^2+^/CaM binding to the receptor [67]. Of interest, and perhaps relevant to understand the functional importance of the cytosolic juxtamembrane region of the EGFR and ErbB2 for their activation, is the fact that no point mutations in/or rearrangement of this region were found in a preliminary study with a limited number (89 cases) of human astrocytic glioma biopsies [68]. After these earlier reports, to our knowledge, no further information on the direct binding of Ca^2+^/CaM to ErbB2 and ErbB3 or ErbB4 has been published.

## 4. Potential Role of CaM on ErbB Receptor Nuclear Translocation

An interesting development on the functionality of ErbB receptors has been the recognition that the full-length receptor can be translocated to the nucleus, exerting transcriptional regulation, e.g., EGFR [69,70,71] and ErbB2 [72]. In the case of ErbB4, it is only the cytosolic domain which is translocated to the nucleus after γ-secretase cleavage [73], where it also exerts transcriptional regulation after association to the transcriptional co-regulator YAP (Yes-associated protein) [74]. The full-length ErbB3 also moves to the nucleus, and it has been localized and associated with the nucleoli [75]. 

CaM may play a role in regulating the nuclear translocation of ErbB receptors (reviewed in [76]). In the case of the EGFR, the cytosolic juxtamembrane region RRRHIVRKRTLRR (residues 645–657) was proposed to be a nuclear localization sequence (NLS) [71,77]. This was later demonstrated using a GFP (green fluorescent protein) construct with the 645–657 sequence added, showing that it was translocated to the nucleus [78]. Using mutant versions of this sequence, the importance of the basic residues (R and K) in the three basic clusters of this region was demonstrated to be essential for the nuclear translocation of the construct [78]. This NLS sequence is coincident, to a great degree, with the CaM-binding site of the receptor RRRHIVRKRTLRRLLQ (residues 645–660) previously described [22]. In the case of ErbB3, a short potential NLS located in the C-terminal tail (residues 1183–1186) was first proposed [75]. However, Hsu and Hung indicated that the NLS of the EGFR is mostly conserved in all the ErbB receptors, including ErbB3 [78]. In the case of ErbB2, it was demonstrated that the sequence KRRQQKIRKYTMRR (residues 655–668) is, indeed, a NLS, as a GFP construct containing this sequence is translocated to the nucleus, and mutation of the basic residues prevented its nuclear translocation [79].

The functional role of CaM on the nuclear translocation of the EGFR has not yet been experimentally ascertained. However, it is conceivable that the binding of Ca^2+^/CaM to the receptor may prevent its nuclear translocation. In any event, further work should be undertaken in this area to establish the actual role of CaM in the translocation of the EGFR into the nucleus.

Of medical interest, tumor treatment with the chemotherapeutic agent cisplatin, a compound that crosslinks purine bases forming adducts in the DNA preventing its repair in case of mishaps (reviewed in [80]), induced the nuclear localization of the EGFR, increasing DNA-dependent protein kinase (DNA-PK) activity and DNA repair and contributing, in this manner, to cisplatin resistance [81,82]. However, mutating the receptor NLS and preventing its nuclear translocation reversed this effect, while the ectopic insertion of the NLS from the large T antigen of the simian virus 40 (SV40) to the C-terminal of the EGFR induced again resistance to cisplatin [81]. The phosphorylation of Thr654, located in the NLS/CaM-BD of the EGFR, by PKCε was required for the translocation of the receptor into the nucleus induced by ionizing radiation [83]. This is of great interest because CaM was previously shown to prevent Thr654 phosphorylation by PKC [22], as mentioned above, and this suggests that the binding of Ca^2+^/CaM to the EGFR may, indeed, prevent the nuclear translocation of the receptor as we hypothesized above.

## 5. ErbB Receptor Signaling and CaM-Dependent Protein Kinases

In addition to the regulation of ErbB receptors through direct interaction with Ca^2+^/CaM, the phosphorylation of these receptors by CaM-dependent kinases also plays a significant role. In this context, the phosphorylation of the EGFR by Ca^2+^/CaM-dependent protein kinase II (CaMK-II) at Ser744, Ser1046/1047, Ser1057 and Ser1142 results in desensitization of the receptor, and the mutation of these sites keeps the receptor active for longer time, favoring interaction with its substrates and, therefore, increasing its oncogenic potential [84,85,86]. Moreover, CaMK-II also phosphorylates ErbB2 at Thr1172, desensitizing the receptor, as a Thr1172Ala point mutation induces the sustained autophosphorylation/activation of this receptor [87].

The activation of the EGFR also results in the activation of CaM-dependent kinase kinase 2 (CaMKK2) by the Ca^2+^/CaM complex, formed upon a transient increase in the cytosolic Ca^2+^ concentration induced by the ligand-dependent activation of the receptor [88]. The translocation of active CaMKK2 to the nucleus results in the expression of different EGFR target genes (e.g., phosphofructokinase, urokinase) by a mechanism not yet fully understood [88]. Moreover, CaMKK2 directly phosphorylates Akt (protein kinase B) at Thr308 in addition to its canonical phosphorylation by PDK1 (phosphoinositide-dependent kinase-1), and the activation of the kinase activity of mTORC2 (mammalian target of rapamycin complex 2), which phosphorylates Akt at Ser473, results in the increased survival of tumor cells [88].

The endogenous CaMK-II inhibitor 1 (CAMK2N1) acts as a tumor suppressor, inhibiting ErbB2 downstream signaling pathways and hence cell proliferation and other cellular functions. Consequently, the decreased expression of this physiological inhibitor, observed in castration-resistant human prostate cancers, prevents ErbB2 desensitization, and this positively correlates with their enhanced growth capacity, invasiveness and poor prognosis [89].

The pregnancy-upregulated non-ubiquitous calmodulin kinase Pnck [90], also denoted as calmodulin-dependent kinase Iβ2 (CaMK-Iβ2) with structural similarities to CaMK-I, is overexpressed in different tumors [91,92,93,94,95], including renal, nasopharyngeal, hepatocellular and breast carcinomas, and provably among other cancer types, increasing their aggressiveness and poor prognosis. As in other tumors, Pnck is co-overexpressed with amplified ErbB2 in more than 30% of human estrogen receptor-negative breast carcinomas [94]. Using the human breast cancer cell line SK-BR-3, it was demonstrated that the expression of Pnck accelerated the progression of the cell cycle and increased the proliferation rate of these cells in addition to inducing enhanced cell survival and resistance to trastuzumab, a monoclonal antibody against ErbB2 used in the clinic [94]. The increased proliferation and in vitro resistance to trastuzumab mediated by Pnck was partially reverted by knocking down PTEN (phosphatase and tensin homolog), although no clear explanation of this paradoxical effect was given; this phosphatidylinositol-3,4,5-trisphosphate 3-phosphatase mostly acts as a tumor suppressor, although the authors of this work hypothesized the possibility that Pnck could augment the activity of residual PTEN [94] but without providing any experimental evidence. Pnck is also involved in initiating the degradation of the EGFR in a ligand-independent mode [96]. It was proposed by the same group as an hypothetical mechanism of this process, in which the Pnck-mediated phosphorylation of Hsp90 (heat shock protein 90) bound to the EGFR induced the detachment of this chaperone from the receptor, followed by the proteolysis of the liberated EGFR via the proteasome–lysosomal pathways [97].

## 6. CaM Function on ErbB Receptor Signaling Pathways

The ligand-dependent activation of the EGFR and ErbB2 using heterodimerization with ErbB3 upon neuregulin binding results in the CaM-mediated activation of the downstream kinase Akt. In estrogen receptor-negative human breast carcinoma cells usually overexpressing ErbB2, it was demonstrated that the addition of EGF induced the translocation of Akt and CaM to the plasma membrane, where the activation of Akt occurs. Akt activation was abolished by the CaM antagonists W-7 or by downregulating the three CaM genes using siRNAs but not in estrogen receptor-positive cells, which is indicative of the presence of an alternative CaM-independent pathway in these cells [98]. In addition, we noticed that the addition of the CaM antagonist W-7 to breast adenocarcinoma SK-BR-3 cells induced the concomitant inhibition of ErbB2 and the Akt and ERK1/2 (extracellular-regulated kinases 1 and 2) pathways as expected, while the phosphorylation (activation) of CREB (cAMP response element-binding protein) and ATF1 (activating transcription factor-1) increased, most likely due to the absence of the CaM/calcineurin-mediated dephosphorylation of these transcription factors [66].

Notwithstanding the canonical CaM-dependent phospho-Ser/Thr-directed activity of calcineurin (reviewed in [99]), an old report described that this phosphatase was able to dephosphorylate phospho-Tyr-casein/histone prepared in the presence of EGF with a membrane fraction from human epidermoid A431 tumor cells [100], which is a cell line that overexpresses the EGFR. This report, however, does not demonstrate that calcineurin could dephosphorylate the activated EGFR and/or substrates of this receptor in a physiological cellular setting.

The CaM-binding scaffold protein IQGAP1 (Ras-GTPase-activating-like protein 1) binds in a Ca^2+^-independent manner to different components of the mitogen-activated protein kinase (MAPK) pathway downstream of the EGFR but also directly interacts with the receptor. In HeLa cells, this results in IQGAP1 phosphorylation at Ser1443 by PKCα (protein kinase Cα) because this downstream kinase is activated by the receptor upon EGF addition [101].

Treating human lung adenocarcinoma cells with the EGFR tyrosine kinase inhibitor gefitinib, used as a chemotherapeutic agent in the clinic and known with the commercial name Iressa, inhibits cell proliferation and reduces cell viability. This has been shown to be due, at least in part, by inducing the activation of the CaM-dependent eukaryotic elongation factor-2 kinase (eEF2K), as downregulating this Ser/Thr-kinase with siRNA or a chemical agent facilitated the proliferation of these tumor cells [102].

It is well known that the EGFR can be phosphorylated by c-Src at certain tyrosine residues [103,104]. However, reciprocally, the sustained ligand-dependent activation of the EGFR is able to activate the tyrosine kinase c-Src [105]. We confirmed these results demonstrated in A431 tumor cells, showing that this activation was inhibited by the CaM antagonist W-7, as expected [106], due to the activating effect of Ca^2+^/CaM on the receptor, as described above. However, in addition, we also demonstrated that Ca^2+^/CaM and Ca^2+^-free CaM both directly bind and enhance c-Src activity; these processes are also inhibited by W-7 [106]. This represents a reciprocal curious cross-talk between two CaM-regulated tyrosine kinases, EGFR and c-Src.

**Figure 4:**
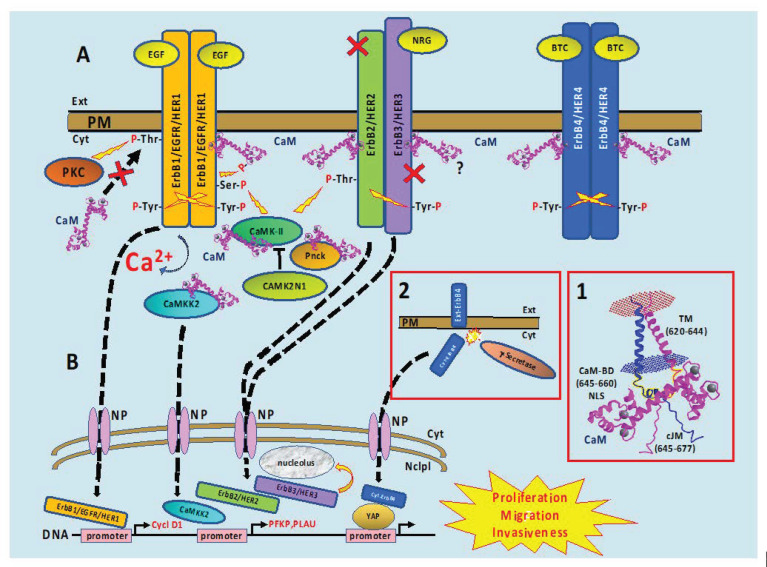
Direct and indirect regulation of the ErbB receptors by CaM. (**A**) The drawing shows the ErbB1/EGFR/HER1 homodimer, ErbB2/HER2:ErbB3/HER3 heterodimer and ErbB4/HER4 homodimer in the plasma membrane, activated by auto(trans)phosphorylation (yellow lightening) upon binding of the ligands: EGF, NRG and BTC, respectively. The red crosses in the ErbB2:ErbB3 heterodimer indicate the absence of ligand binding capacity of ErbB2 and absence of tyrosine kinase activity of ErbB3. The drawing in *inset 1* depicts an enlarged view of Ca^2+^/CaM bound to the CaM-BD (highlighted in yellow) in the cytosolic juxtamembrane region of an EGFR homodimer. The NLS sequence coincides, in part, with the CaM-BD sequence. The numbers indicate the amino acid residues in the sequence of the mature EGFR. The region of CaM interacting with the receptor is arbitrarily selected. The structures of Ca^2+^-bound CaM (PDB ID: 1CLL) [32] and the transmembrane–cytosolic juxtamembrane segment of the EGFR (PDB ID: 2M20) [107] were taken from the National Center for Biotechnology Information (NCBI). The transient increase in the cytosolic Ca^2+^ concentration generated by ligand-dependent EGFR activation [108] induces the formation of the Ca^2+^/CaM complex and activation of CaMK-II, CaMKK2 and Pnck. Ca^2+^/CaM is shown bound to the ErbB receptors and CaMK-II, CaMKK2 and Pnck. The question mark in ErbB3 indicates that Ca^2+^/CaM has a very low affinity for this receptor; therefore, its physiological function is questionable. The tumor suppressor CAMK2N1 is an inhibitor of CaMK-II, which inhibits ErbB2 downstream signaling pathways. The EGFR phosphorylates CaM at Tyr99 and Tyr138, which interacts with the receptor at the same site as non-phosphorylated CaM; more details about this process are shown in Figure 1. The phosphorylation of Thr654 by PKC prevents Ca^2+^/CaM binding to the EGFR (red cross). CaMK-II phosphorylates serine and threonine residues in the EGFR and ErbB2, respectively, inducing desensitization of the receptors. (**B**) The full length EGFR, and perhaps the full length ErbB2 and ErbB3, are translocated to the nucleus. ErbB3 has been located at the nucleoli. ErbB4, however, is previously proteolyzed by γ-secretase (*inset 2*) releasing its cytosolic segment, which is translocated to the nucleus. The ErbB receptors exert in the nucleus transcriptional functions, contributing to the enhanced proliferation, migration and invasiveness of tumor cells. For additional signaling pathways and details, see the text. BTC, betacellulin; CaM, calmodulin; CaM-BD, CaM-binding domain; CaMK-II, CaM-dependent protein kinase II; CAMK2N1, CaMK-II inhibitor 1; CaMKK2, CaM-dependent kinase kinase 2; cJM, cytosolic juxtamembrane segment; Cycl D1, cyclin D1; Cyt, cytosol; Cyt-ErbB4, cytosolic ErbB4 region; EGF, epidermal growth factor; Ext, extracellular medium; Ext-ErbB4, extracellular ErbB4 region; Nclpl, nucleoplasm; NLS, nuclear localization sequence; NP, nuclear pore; NRG, neuregulin; PFKP, phosphofructokinase; PKC, protein kinase C; PM, plasma membrane; PLAU, plasminogen activator urokinase; Pnck, pregnancy-upregulated non-ubiquitous calmodulin kinase; TM, transmembrane segment; YAP, yes-associated protein.

Considering the multiple points of involvement of CaM and CaM-BPs in the direct and indirect regulation of the ErbB receptors and to briefly summarize the most significant features, Figure 4 depicts some of the major facts covered in this article.

## 7. Concluding Remarks

Considering the naturally occurring CaM mutants [7,13,14,15,16]), it would be of interest to investigate whether these mutants bind and negatively affect the activation of the ErbB receptors, particularly the EGFR and provably ErbB2. If so, at least some of the CaM mutants could be considered potential tumor suppressors.

The ErbB receptors play an essential role in the development of different organs, for example, the nervous system and the heart (reviewed in [109,110,111]), and it may be expected that the occurrence of CaM mutants may also induce failure of normal embryonic development by this mechanism. Moreover, the activity of ErbB receptors could be indirectly affected by any negative action of CaM mutants on CaMK-II [84,85,86,87]. In this context, it has been shown that introducing the arrhythmogenic CaM mutation D130G in the *CALM2* gene of mouse embryonic carcinoma P19CL6 cells and allowing these cells to differentiate into cardiomyocytes, this procedure decreased their spontaneous beat frequency because of the loss of CaMK-II function [112]. For an analogy, this suggests that this CaM mutant could likewise prevent CaMK-II-mediated desensitization of the EGFR and ErbB2 (see [84,85,86,87]) and, therefore, increase their carcinogenic potential.

Due to gene rearrangement, the loss of exons 2–7 of the EGFR coding part of its ectodomain yields the expression of a mutant receptor denoted as EGFRvIII, which lacks ligand binding capacity. This mutant receptor is commonly expressed in some tumors, including glioblastomas, inducing enhanced tumor progression (reviewed in [113]). An interesting topic for future research could be to investigate whether wild-type CaM and its mutated forms show any implications in EGFRvIII signaling.

In addition, although in limited preliminary studies, no mutations in the CaM-BD of the EGFR were found in human astrocytic gliomas [68,114], further studies should be conducted in this regard in different tumors to study any potential functional implications that these mutations may have.

## Data Availability

Not applicable.
